# Insulin-Producing Cells Generated from Dedifferentiated Human Pancreatic Beta Cells Expanded In Vitro

**DOI:** 10.1371/journal.pone.0025566

**Published:** 2011-09-30

**Authors:** Holger A. Russ, Elad Sintov, Leeat Anker-Kitai, Orr Friedman, Ayelet Lenz, Ginat Toren, Chen Farhy, Metsada Pasmanik-Chor, Varda Oron-Karni, Philippe Ravassard, Shimon Efrat

**Affiliations:** 1 Department of Human Molecular Genetics and Biochemistry, Sackler School of Medicine, Tel Aviv University, Tel Aviv, Israel; 2 Bioinformatics Unit, George Wise Faculty of Life Sciences, Tel Aviv University, Tel Aviv, Israel; 3 Department of Biotechnology and Biotherapy, Hôpital Pitié Salpêtrière, Paris, France; Boston University, United States of America

## Abstract

**Background:**

Expansion of beta cells from the limited number of adult human islet donors is an attractive prospect for increasing cell availability for cell therapy of diabetes. However, attempts at expanding human islet cells in tissue culture result in loss of beta-cell phenotype. Using a lineage-tracing approach we provided evidence for massive proliferation of beta-cell-derived (BCD) cells within these cultures. Expansion involves dedifferentiation resembling epithelial-mesenchymal transition (EMT). Epigenetic analyses indicate that key beta-cell genes maintain open chromatin structure in expanded BCD cells, although they are not transcribed. Here we investigated whether BCD cells can be redifferentiated into beta-like cells.

**Methodology/Principal Finding:**

Redifferentiation conditions were screened by following activation of an insulin-DsRed2 reporter gene. Redifferentiated cells were characterized for gene expression, insulin content and secretion assays, and presence of secretory vesicles by electron microscopy. BCD cells were induced to redifferentiate by a combination of soluble factors. The redifferentiated cells expressed beta-cell genes, stored insulin in typical secretory vesicles, and released it in response to glucose. The redifferentiation process involved mesenchymal-epithelial transition, as judged by changes in gene expression. Moreover, inhibition of the EMT effector SLUG (SNAI2) using shRNA resulted in stimulation of redifferentiation. Lineage-traced cells also gave rise at a low rate to cells expressing other islet hormones, suggesting transition of BCD cells through an islet progenitor-like stage during redifferentiation.

**Conclusions/Significance:**

These findings demonstrate for the first time that expanded dedifferentiated beta cells can be induced to redifferentiate in culture. The findings suggest that ex-vivo expansion of adult human islet cells is a promising approach for generation of insulin-producing cells for transplantation, as well as basic research, toxicology studies, and drug screening.

## Introduction

Beta-cell replacement is a promising approach for the cure of type 1 diabetes, however, its application is limited by the shortage of pancreas donors. In-vitro expansion of human cadaveric islet beta cells represents an attractive strategy for generation of abundant beta-like cells [1]–[5]. Human beta cells manifest a very low proliferation capacity in vivo [6]–[8], and intact isolated islets cultured in suspension do not proliferate, although they remain functional for months [9]. When islets are allowed to attach, limited replication of beta cells can be induced by growth factors or extracellular matrix components [10] before the beta-cell phenotype is lost. To determine the fate of cultured beta cells we established a lineage tracing system based on a dual lentiviral vector system [11]. This system provided evidence for survival and dedifferentiation of adult human beta cells, and significant replication of beta-cell-derived (BCD) cells. The phenotypic changes in BCD cells resembled epithelial-mesenchymal transition (EMT) [12], as originally suggested by Gershengorn et al. [2]. EMT likely results from islet cell dissociation and exposure to culture conditions, and may be involved in triggering changes in gene expression, leading to beta-cell dedifferentiation and replication. Epigenetic analyses of BCD cells indicated that key beta-cell genes maintained a partially open chromatin structure, although they were not transcribed [13]. This epigenetic memory was maintained even following BCD cell reprogramming into induced pluripotent stem (iPS) cells [13]. We hypothesized that this epigenetic memory rendered BCD cells attractive candidates for generation of insulin-producing cells by redifferentiation. BCD cell expansion could generate sufficient cells for human beta-cell replacement, if the lost cell phenotype can be restored. Here we report that BCD cells can be preferentially redifferentiated by a combination of soluble factors in serum-free medium (SFM). The redifferentiated cells re-express beta-cell genes, process and store insulin in typical secretory vesicles, and release it in response to glucose. The redifferentiation process involves mesenchymal-epithelial transition (MET) and activation of genes expressed in islet progenitor cells. These findings suggest that ex-vivo expansion of adult human islet beta cell is a promising approach for generation of insulin-producing cells for transplantation.

## Materials and Methods

### Ethics statement

This study was conducted according to the principles expressed in the Declaration of Helsinki. The Institutional Review Boards of the following medical centers, which provided human islets, each provided approval for the collection of samples and subsequent analysis: University of Geneva School of Medicine; San Raffaele Hospital, Milan; Faculty of Medicine, Lille 2 University; Massachusetts General Hospital; Washington University; University of Pittsburgh; Scharp/Lacy Institute; University of Minnesota. All donors provided written informed consent for the collection of all samples and subsequent analysis.

### Cell culture

Islets from individual donors (Table 1) were dissociated into single cells, and beta cells were labeled and expanded as previously described [4], [11], [12], [14]. For differentiation cells were trypsinized and seeded at 3.2×10^4^/cm^2^ in ultra-low attachment plates (Corning). SFM consisted of CMRL 1066 containing 5.6 mM D-glucose and supplemented with 1% BSA fraction V (Sigma), ITS (Gibco-Invitrogen), penicillin (50 units/ml), and streptomycin (50 µg/ml). Redifferentiation Cocktail (RC) treatment consisted of 6 days in SFM supplemented with 25 mM D-glucose, N2 and B27 supplements (Stem Cell Technologies), 10 mM nicotinamide (Sigma), 8 nM exendin-4 (Acris), and 8 nM activin A (Peprotech), followed by 2 days in the same medium lacking activin A and containing a reduced glucose concentration (5.6 mM). Human fibroblasts were maintained in DMEM containing 10% FCS (Gibco), 100 U/ml penicillin, and 100 µg/ml streptomycin. Human bone marrow-derived mesenchymal stem cells (BM-MSC) were isolated and cultured as described [15].

**Table 1:** Donors of islets used in the study.

Donor No.	Donor Sex	Donor Age (y)	Donor BMI	Islet purity (%)
1	F	27	ND	95
2	F	63	39.3	80
3	F	66	29.4	83
4	M	50	37.0	92
5	F	34	22.5	80
6	M	42	22.5	70
7	F	24	30.9	90
8	M	57	34.3	75
9	M	69	30.4	85
10	M	45	31.6	ND
11	M	54	26.9	85
12	M	47	23.0	85
13	F	51	39.3	80
14	M	ND	35.0	80
15	F	36	43.0	90
16	M	26	19.8	65
17	M	57	32.4	80
18	F	69	29.7	90
19	M	29	20.7	80
20	M	38	22.9	85
21	M	58	33.4	65
22	F	55	26.6	80
23	M	55	22.4	70
24	F	38	24.0	75
25	M	55	27.0	70
26	M	62	24.2	90
27	F	38	20.8	95
28	M	62	30.6	ND
29	F	44	21.5	85
30	M	40	29.0	95
31	F	47	33.2	70
32	F	50	42.3	85
33	M	21	33.8	85
34	M	62	31.8	99
35	M	44	24.7	99
36	M	27	19.0	85
37	M	55	22.4	70
38	F	38	24.0	75
39	F	62	27.1	95
40	M	59	23.0	85
41	M	22	19.8	80
42	M	39	27.4	98
**Average±SD**		**47±14**	**28±6**	**83±9**

### Virus production and cell transduction

RIP-Cre/ER and pTrip–loxP-NEO-STOP-loxP-eGFP lentivirus vectors have been described [12]. *SLUG* shRNAs (accession numbers TRCN-15389, - 15388, - 15390, - 15391, -15392), and a non-target shRNA, all cloned in the pLKO.1 lentiviral vector, containing a puromycin-resistance gene, were obtained from the RNAi Consortium (Sigma-Aldrich). Virus production, cell infection, and tamoxifen treatment were previously described. For SLUG inhibition, transduced cells were selected for puromycin resistance (1 µg/ml) for 3 days.

### Cell sorting

Labeled cells were sorted using a FACS Aria sorter (Becton Dickinson, San Jose, CA) as described [11], [12].

### Flow cytometry

Cells were dissociated with trypsin, resuspended in PBS containing 2% FCS, and filtered through a cell strainer. Flow cytometry was preformed on FACsort (BD Bioscience) using FlowJo software (Tree Star). Final analysis was done using Cyflogic software (Cyflogic). Untreated cells, and infected cells without differentiation treatment, served as controls.

### Cell proliferation and apoptosis analyses

Cells were incubated with 10 µM BrdU (Sigma) for 7 days, starting one day following initiation of RC treatment. Apoptotic cells was detected by TUNEL staining using the TACS•XL Apoptosis Detection Kit (R&D Systems Inc., Minneapolis, MN).

### qPCR analyses

Total RNA was extracted using RNAeasy Plus Micro Kit (Qiagen) or Trizol (Sigma-Aldrich) and treated with DNA-Free™ (Ambion). cDNA was prepared using High Capacity cDNA RT Kit (Applied Biosystems). qPCR was carried out in triplicates using TaqMan Universal PCR Master Mix (Applied Biosystems) or Universal Probe Library Master Mix (Roche) in 7300 Real-time PCR System (Applied Biosystems). Results were normalized to glyceraldehyde-3-phosphate dehydrogenase (GAPDH) and/or human large ribosomal protein (RPLPO) transcripts. Primer sequences are listed in Table 2. All reactions were performed with annealing at 60°C for 40 cycles. For undetectable transcripts, the cycle number was set to 40 for comparisons.

**Table 2:** Primer sequences.

Gene	Sense primer	Antisense primer
INS	Taqman probe Hs 00355773 m1	Taqman probe Hs 00355773 m1
	agaagaggccatcaagcaga	caggtgttggttcacaaagg
PDX1	Taqman probe Hs_00426216_m1	Taqman probe Hs_00426216_m1
	aagctcacgcgtggaaag	gccgtgagatgtacttgttgaa
FOXA2	Taqman probe Hs_00232764_m1	Taqman probe Hs_00232764_m1
MAFA	Taqman probe Hs01651425_s1	Taqman probe Hs01651425_s1
	agcgagaagtgccaactcc	ttgtacaggtcccgctcttt
GCG	Taqman probe Hs01031536_m1	Taqman probe Hs01031536_m1
	gtacaaggcagctggcaac	tgggaagctgagaatgatctg
SST	Taqman probe Hs00174949_m1	Taqman probe Hs00174949_m1
	accccagactccgtcagttt	acagcagctctgccaagaag
PPY	Taqman probe Hs00237001_m1	Taqman probe Hs00237001_m1
	tctagtgcccatttactctggac	gcaggtggacaggagcag
AMY2A	Taqman probe Hs00420710_g1	Taqman probe Hs00420710_g1
GAPDH (h)	Taqman probe Hs_99999905_m1	Taqman probe Hs_99999905_m1
	agccacatcgctcagacac	gcccaatacgaccaaatcc
RPLPO	Taqman probe Hs_99999902_m1	Taqman probe Hs_99999902_m1
	tctacaaccctgaagtgcttgat	caatctgcagacagacactgg
SUR1	agaccctcatgaaccgacag	ggctctgtggcttttctctc
GCK	gcagatgctggacgacag	tcctgcagctggaactctg
KIR6.2	tgtgtcaccagcatccactc	cacttggacctcaatggagaa
IAPP	ttaccaaattgtagaggctttcg	ccctgcctctatacactcactacc
PC1/3	tgatcccacaaacgagaaca	tgtgattatttgcttgcatgg
HLXB9	Taqman probe Hs00907365_m1	Taqman probe Hs00907365_m1
NEUROD1	ctgctcaggacctactaacaacaa	gtccagcttggaggacctt
NKX2-2	cgagggccttcagtactcc	ggggacttggagcttgagt
NKX6-1	Taqman probe Hs00232355_m1	Taqman probe Hs00232355_m1
	cgttggggatgacagagagt	cgagtcctgcttcttcttgg
SNAI1	gctgcaggactctaatccaga	atctccggaggtgggatg
SNAI2	tggttgcttcaaggacacat	gttgcagtgagggcaagaa
NCAD	ctccatgtgccggatagc	cgatttcaccagaagcctctac
ECAD	gccgagagctacacgttca	gaccggtgcaatcttcaaa
SOX9	gtacccgcacttgcacaac	tcgctctcgttcagaagtctc
PTF1A	Taqman probe Hs_00603586_g1	Taqman probe Hs_00603586_g1
ARX	gcaccacgttcaccagcta	cagcctcatggccagttc
TCF2	Taqman probe Hs_00172123_m1	Taqman probe Hs_00172123_m1
TWIST	aaggcatcactatggactttctct	gccagtttgatcccagtatttt
PAX4	accccacctaaagcctgtct	aggcaaagcagtcctgagtc
INSM1	cgctgtgttcatggtctagaaa	catagagagcagagattggtaggc
KRT19	gccactactacacgaccatcc	caaacttggttcggaagtcat

### DNA microarray analysis

Hybridization to Affymetrix GeneChip Human Gene 1.0 ST Arrays, washing, and scanning were performed according to the manufacturer (Affymetrix, Santa Clara, CA). Microarray analysis was performed on CEL files using Partek® Genomics Suite TM (Partek Inc., St. Louis, MO). Data were normalized with the multi-average method [16]. Batch effect removal was applied for the different samples, followed by one-way ANOVA. Clustering analysis was performed by Partek Genomics Suite software with Pearson's dissimilarity correlation by average linkage methods. All data is MIAME compliant. The raw data has been deposited in the GEO database (accession number GSE30732).

### ****Transmission electron microscopy

Cells were fixed in 2.5% glutaraldehyde (EMS) in PBS overnight at 4°C and postfixed in 1% OsO4 (EMS) in PBS for 2 hours at 4°C. Dehydration was carried out in graded ethanol solutions, followed by embedding in Glycid ether. Thin sections were mounted on Formvar/Carbon coated grids and examined in Jeol 1200EX transmission electron microscope. Images were captured using SIS Megaview III camera and iTEM imaging platform (Olympus).

### Immunofluorescence analyses

Cells were trypsinzed, spotted on slides using a Shandon Cytospin4 centrifuge (Thermo Scientific), and fixed for 15 min at RT in 4% paraformaldehyde (PFA). Paraffin sections were rehydrated, and antigen retrieval was preformed before staining. For BrdU staining slides were incubated in 1.5 M HCl for 20 min, followed by 0.1 M sodium borate (pH 8,5) for 10 min at RT and 3 washes in PBS. All samples were blocked for >10 min in blocking buffer (1% BSA, 5% fetal goat serum, and 0.2% saponin). Primary antibodies (Table 3) were diluted in blocking buffer, and samples were incubated for either 1 hour at RT or overnight at 4°C. Slides were washed in PBST and incubated for 40 min with secondary antibody conjugated to Alexa fluorophores (all from Invitrogen, 1∶1000). Images were taken using a Leica SP5 confocal microscope. Images of fluorescent living cells were taken with a Nikon microscope equipped with a NIS Elements camera and software. DNA was stained with DAPI (Sigma).

**Table 3:** Antibodies used in immunofluorescence.

Antigen	Antibody species	Source	Dilution
eGFP	mouserabbit	ChemiconInvitrogen	1∶5001∶1000
C-peptide (human)	mouserabbitrat	BiodesignAbcamBCBC	1∶2001∶1001∶1000
Glucagon	mouse	Sigma	1∶2000
Somatostatin	rabbit	Dako	1∶400
PPY	rabbit	Dako	1∶200
Ki67 (human)	rabbit	Zymed	1∶50
Vimentin	mouse	Calbiochem	1∶50
PDX1 (human)	rabbit	Abcam	1∶500
PCSK1	mouse	Millipore	1∶100
IAPP	mouse	Thermo Scientific	1∶30
NKX2.2	mouse	Hybridoma Bank	1∶1000
NKX6.1	mouse	Gift from P. Serup	1∶1000
SOX9	rabbit	Chemicon	1∶200
FOXA2	rabbit	Abcam	1∶1000
BrdU	mouse	Chemicon	1∶100

### Immunoblotting

Total protein was extracted for 10 min in 1% NP40 containing protease inhibitor cocktail. 20 µg protein were resolved on SDS-PAGE gel and electroblotted onto Immobilon-P membrane (Milipore), followed by incubation with rabbit anti-SOX9 (Chemicon, 1∶200) or mouse anti-SLUG (Sigma, 1∶50). Mouse anti-actin (Sigma, 1∶2000) or mouse anti-HSC70 (SantaCruz, 1∶1000) was used to monitor loading. The bound antibody was visualized with horseradish peroxidase-conjugated anti-IgG and SuperSignal West Pico Chemiluminescent Substrate (Pierce). Quantification was done using TINA software.

### In situ hybridization

In situ hybridization analysis was performed on paraffin sections of fetal pancreas and frozen sections of cell clusters as described [17]. Cell clusters were fixed overnight in 4% PFA in PBS at 4°C, incubated in 30% sucrose in PBS for 48 hours at 4°C, embedded in OCT, and snap-frozen. Nine-µm sections were cut using a CM3050S cryostat (Leica). A 557-bp fragment from the first exon of *NGN3* was amplified from human genomic DNA using the primers 5′agggagaagcagaaggaacaag3′ and 5′cctaagagcgagttggcactg3′ and cloned into pGEM-T Easy Vector system (Promega). Sense/antisense probes were labeled with digoxigenin using Sp6 or T7 polymerases, and hybridized at 6 µg/ml overnight at 65°C. Slides were treated with RNaseA, washed, blocked with 20% normal goat serum, and incubated with sheep anti-digoxigenin Fab fragments conjugated to alkaline phosphatase (1∶2500, Roche) in blocking solution overnight at 4°C. Slides were washed, and pigment was developed using BM Purple (Roche).

### Insulin content and secretion

Cell were preincubated for 1 hour in Krebs–Ringer buffer (KRB), followed by incubation for 2 hours in KRB containing 0.5 mM 1-isobutyl 3-methylxanthine (IBMX) and 16.7 mM glucose. C-peptide content was determined in acidic alcohol cell extract. Human C-peptide levels were quantified using an ultrasensitive ELISA kit (Mercodia; sensitivity 1.5 pmol/L; cross-reactivity with insulin and proinsulin <0.0006% and <1.8%, respectively). Human proinsulin levels were quantified using a Proinsulin ELISA kit (Mercodia; sensitivity 0.5 pmol/L).

### Statistical analysis

Significance was determined using two-tailed t-test and χ^2^ test. To approach a normal distribution, logarithmic transformation was preformed.

## Results

### Development of conditions for BCD cell redifferentiation

**Figure 1:**
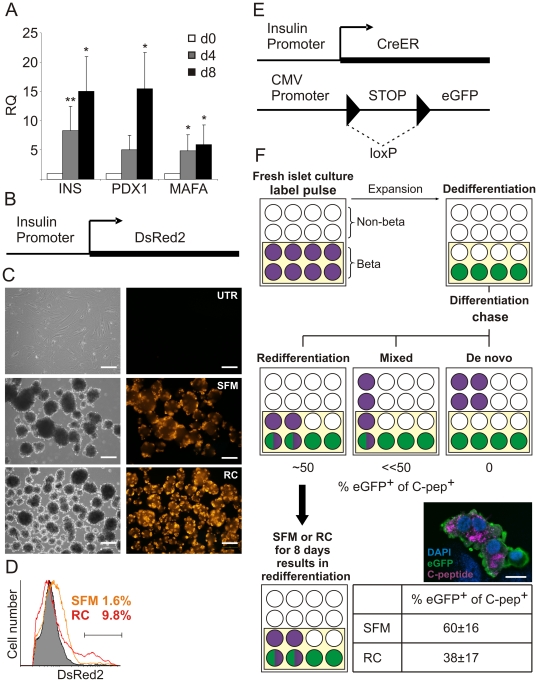
Development of conditions for islet cell differentiation, and demonstration that differentiation represents predominantly BCD-cell redifferentiation. **A:** qPCR analysis of beta-cell gene expression in expanded islet cells at p6 incubated in SFM for the indicated number of days. Values are mean±SD relative to untreated cells (d0) (n = 3–4 donors) and normalized to human RPLPO. *p<0.05; **p<0.005. **B–D:** An assay for screening islet cell differentiation conditions. Islet cells are transduced with a RIP-DsRed2 lentivirus (**B**) followed by testing different differentiation conditions. **C:** No DsRed2 expression is observed in expanded cells at p5 (UTR, untreated cells), while cell transfer to SFM or RC results in insulin promoter activation, which can be monitored in live cells by appearance of DsRed2 fluorescence. Bar = 200 µm. **D:** Flow cytometry quantitation of differentiation reveals that treatment with RC results in a 6-fold higher number of redifferentiated cells, compared with SFM. **E:** Schematic representation of the viral vectors used for beta-cell labeling. **F:** Predictions for generation of insulin-expressing cells by different cell sources within expanded islet cell populations. Initial cultures of islet cells consist of approximately half insulin-producing beta cells (purple filled circles) and half non-beta cells (open circles). The labeling efficiency of beta cells is about 50% (green filled circles). Dedifferentiated islet cells are expanded and then differentiated in SFM or RC, and the percent eGFP^+^ cells among C-pep^+^ cells is quantified. Redifferentiation of BCD cells is expected to result in ∼50% co-staining, given the eGFP labeling efficiency. *De novo* differentiation of insulin-expressing cells from non-BCD cells should result in no co-staining, while occurrence of both mechanisms will result in a low percent of co-stained cells. The actual co-staining quantitation (bold arrow) revealed that redifferentiation of BCD cells was the predominant source of insulin-expressing cells generated in both conditions. Percentages indicate fraction of eGFP^+^ cells among C-pep^+^ cells redifferentiated from expanded islet cells at p4–6 (based on counting >400 cells in each of 3 donors). A representative micrograph is shown. DNA was stained blue with DAPI. Bar = 10 µm.

Incubation of expanded islet cells in SFM resulted in cell cluster formation (Figure 1C) and a gradual increase in expression of beta-cell transcripts during the 8-day incubation period (Figure 1A). To screen for soluble factors which induce further differentiation, islet cells were transduced with an insulin promoter-DsRed2 (RIP-DsRed2) reporter lentivirus (Figure 1B). Loss of insulin promoter activity during cell expansion, coupled with DsRed2 half-life of 4.5 days, resulted in marker disappearance (Figure 1C). Following expansion, cells were transferred to SFM containing various agents, and differentiation was evaluated in live cells by scoring fluorescence reappearance. Based on preliminary screening of individual agents and their combinations, a two-step differentiation protocol termed Redifferentiation Cocktail (RC) was optimized (see Materials and Methods). The factors included activin A, exendin-4, nicotinamide, and high glucose concentrations, which have been shown to promote beta-cell differentiation [18]–[21]. N2, B27, and ITS, were included to prevent cell death in the absence of serum. Glucose concentration was reduced in Step 2 to increase cell sensitivity to glucose-stimulated insulin release [22]. This treatment resulted in cell cluster formation similar to that seen with SFM alone (Figure 1C). However, the number of DsRed2^+^ cells was 6-fold higher in RC, compared with SFM (Figure 1D).

Since islet cell cultures represent a mix of several cell types, the observed differentiation could result from redifferentiation of BCD cells, or de-novo differentiation of other cells. To determine the origin of the newly-generated insulin-expressing cells we used our inducible lineage tracing approach (Figure 1E). BCD cells were labeled during the first days of culture with the RIP-Cre/ER and pTrip–loxP-NEO-STOP-loxP-eGFP lentivirus vectors in the presence of tamoxifen (TM) as previously described [12]. As seen in Figure S1, Cre was specifically expressed in C-pep^+^ cells. Labeled islet cells at passages p4–6 were treated with SFM or RC, and stained for human C-peptide and eGFP. Since the average beta-cell labeling efficiency is 57.5±8.9% [11], the expected incidence of C-pep/eGFP-double-positive cells in case of redifferentiation should be close to this value, while de-novo differentiation should result in 0% co-labeling in the absence of TM. The actual incidence of double-positive cells found following SFM treatment was 60±16%, suggesting that redifferentiation was the predominant mechanism (Figure 1F). Redifferentiation rate was relatively low, with 4.7±3.0% of GFP^+^ cells expressing C-peptide (1–2% of total cells, compared with <0.05% in cells incubated in medium with serum). As observed with SFM alone, a large fraction (38±17%) of C-pep^+^ cells co-stained for eGFP following RC treatment, indicating that generation of C-pep^+^ cells under both conditions occurred primarily through redifferentiation of BCD cells. Since the inducible labeling system was shown to have a leakiness of 1.7% in the absence of TM [12], we can not exclude a small contribution from non-BCD cells, transduced by both vectors, to the eGFP^+^ cell pool following differentiation treatments. However, no increase in the fraction of eGFP^+^ cells in the absence of TM was noted following redifferentiation (data not shown).

### Redifferentiation generates beta-like cells

**Figure 2:**
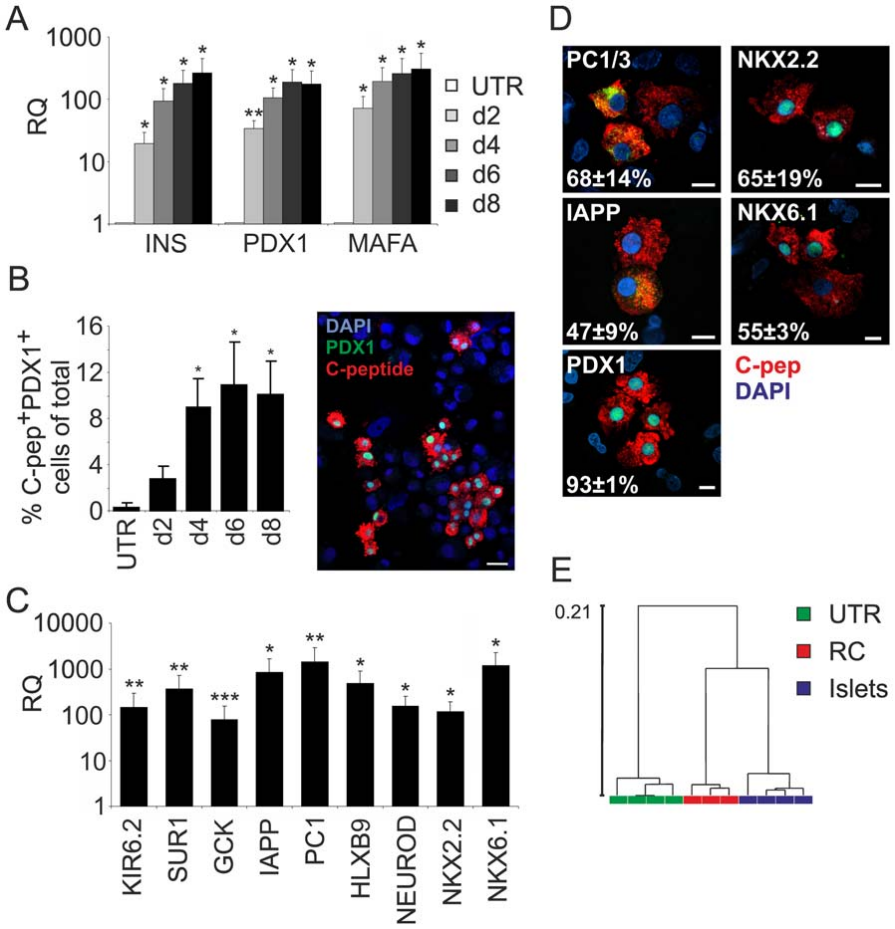
Redifferentiation of BCD cells induced by soluble factors. **A,B:** Kinetics of induction of beta-cell genes in islet cells at p5 treated with RC. **A:** qPCR analysis. Values are mean±SE relative to untreated cells (d0) (n = 3–4 donors) and normalized to human RPLPO or GAPDH. *p<0.05; **p<0.01. **B:** Immunofluorescence analysis of cells co-stained for C-peptide and PDX1. Values are mean±SE (logarithmic scale), based on counting >500 cells from each of 3 donors at each time point. *p≤0.05, relative to untreated cells (d0). A representative micrograph is shown. Bar = 25 µm. **C,D:** Analyses of beta-cell gene expression in islet cells at p5–7 treated with RC for 8d. **C:** qPCR analysis. Values are mean±SE (logarithmic scale) relative to untreated cells (d0) (n = 3–4 donors) and normalized to human RPLPO. *p<0.05; **p<0.005; ***p<0.0005. **D:** Immunofluorescence analysis of cells co-stained for C-peptide and the indicated protein (in green). Bar = 10 µm. The percentages indicate the fraction of cells positive for each marker among C-peptide^+^ cells. Data are mean±SD, based on counting >200 cells from each of 3 donors. **E:** Unsupervised clustering of gene expression patterns detected in cDNA microarray analyses of uncultured islet cells from 4 donors, expanded islet cells at p5 from 4 donors (UTR), and expanded islet cells at p5 from 3 donors treated with RC.

Gene expression analysis of RC-treated cells at different time points revealed a gradual increase in insulin expression (Figure 2A). *PDX1* and *MAFA* transcript levels similarly increased over time (Figure 2A). To determine the kinetics of appearance of C-pep^+^ cells, cells treated with RC were co-stained every other day for C-peptide and PDX1 as markers of mature beta cells. As seen in Figure 2B, the number of double-positive cells increased with time, suggesting stochastic activation of the redifferentiation process, rather than a synchronized initial priming followed by further maturation. The differentiation rate peaked at 10.9±3.7% of total cells on day 6, which is 5–10-fold higher than with SFM alone. Given a fraction of ∼45% BCD cells in the expanded cell population [11], this efficiency represents ∼25% redifferentiation of BCD cells. Transcripts encoding HBLX9, NEUROD, NKX2.2, and NKX6.1 were also strongly induced by RC (Figure 2C). In addition, transcripts encoding KIR6.2, SUR1, GCK, IAPP and PC1/3, were also significantly upregulated. Most redifferentiated cells co-expressed C-peptide and PDX1 (93±1%), NKX2.2 (65±19%), NKX6.1 (55±3%), IAPP (47±9%) and PC1/3 (68±14%) (Figure 2D). Microarray analysis of gene expression showed that RC induced a global phenotypic change in expanded islet cells towards the phenotype of uncultured islet cells, as revealed by hierarchical cluster analyses (Figure 2E). However, the RC-treated samples were still noticeably different from islet cells. This difference likely results from redifferentiation of only part of BCD cells (∼25%) achieved with RC, and from analysis of mixed cell populations. Incubation of primary human fibroblasts or BM-MSCs in RC did not result in detectable insulin or *PDX1* transcripts (data not shown), further supporting the specific redifferentiation capacity of BCD cells.

**Figure 3:**
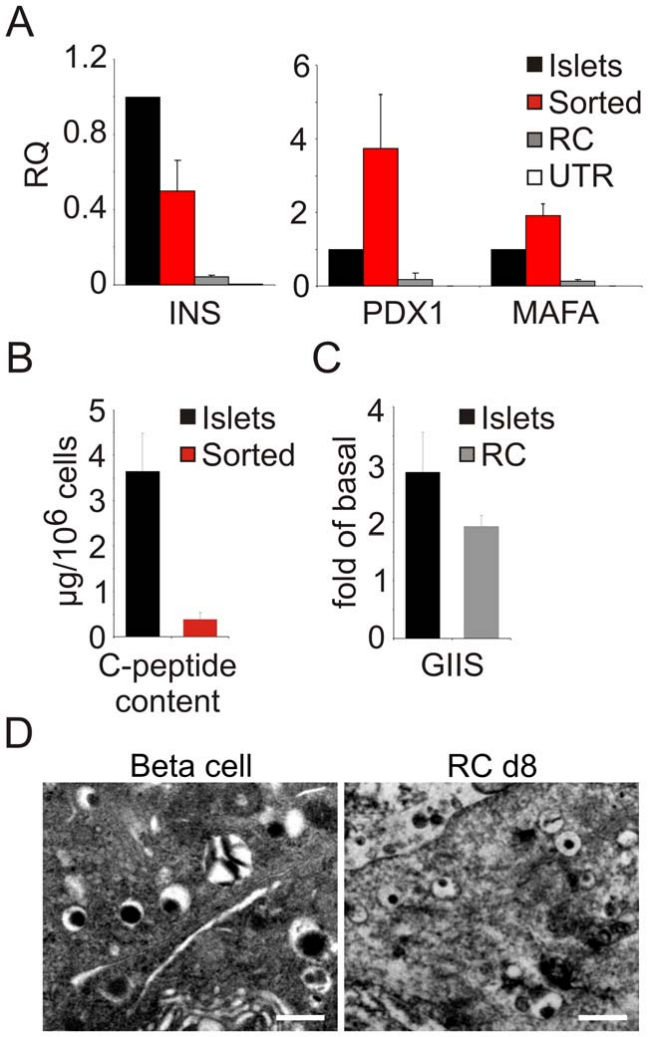
Insulin production and secretion in sorted redifferentiated BCD cells. Expanded islet cells (p6–7) were transduced with RIP-DsRed2 vector and treated with RC for 8d. DsRed2^+^ cells were sorted, and unsorted (RC) and sorted cells were analyzed. **A:** qPCR analysis of beta-cell transcripts. Values are mean±SE, relative to human islets (n = 3 donors) and normalized to human RPLPO. **B:** Human C-peptide content of fresh human islets and DsRed2-sorted RC-treated islet cells. Values are mean±SE (n = 3 donors). Note that most of the insulin stored is processed (only 9% proinsulin). **C:** Glucose-induced insulin secretion from human islets and RC-treated expanded islet cells in the presence of 16.7 mM glucose and IBMX. Values are mean±SD of human C-peptide, relative to 0 mM glucose and IBMX (n = 3 donors). **D:** Electron microscopic analysis reveals the presence of typical insulin secretory vesicles in cells at p6 treated with RC for 8d, comparable to those seen in beta cells in isolated human islets. Bar = 0.5 µm.

To characterize the redifferentiated cells in greater detail, expanded islet cells carrying the RIP-DsRed2 reporter gene were incubated with RC. After 8 days cell clusters were dissociated, and cells were FACS-sorted and characterized. Most DsRed2^+^ cells (>95%) stained for C-peptide. qPCR analysis of sorted DsRed2^+^ cells detected levels of insulin transcripts averaging 50% of those present in human islets (Figure 3A). Given the average islet purity of 83±9% (Table 1), and an average of 55% beta cells in human islets [23], these islet preparations were expected to contain ∼45% insulin-positive cells; therefore insulin transcript levels in sorted cells represent about 40% of those of normal human beta cells. These levels represent a 13,300-fold increase over untreated cells (Figure 3A). The levels of *PDX1* and *MAFA* transcripts were comparable to those in human islets (Figure 3A). In addition, sorted redifferentiated cells stored 10% of the C-peptide levels found in isolated human islets (Figure 3B). Most of the insulin (∼91%) was processed, as judged by human proinsulin-specific ELISA (data not shown). Redifferentiated cells challenged with 16.7 mM glucose and 0.5 mM IBMX showed a 2-fold increase in insulin release, compared with a 3-fold increase seen in islets (Figure 3C). Typical insulin vesicles were detected by electron microscopy, although their dense core appeared smaller, compared with human islets (Figure 3D).

Expanded BCD cells maintain an open chromatin structure even at the latest passage analyzed (p11) [13]. To test if late-passage cells were still capable of redifferentiation they were treated with RC. Islet cell cultures could be induced to efficiently redifferentiate even after a 4096-fold expansion (Figure S2). The residual insulin transcript levels in expanded islet cells declined with passaging. Thus, although RC stimulated a similar fold increase in insulin transcripts at early and late passages, transcript levels induced by RC declined with passaging, when compared with those in islets. One explanation for this observation could be that the epigenetic memory in BCD cells is eroded with each cell division.

### Redifferentiation is associated with MET

**Figure 4:**
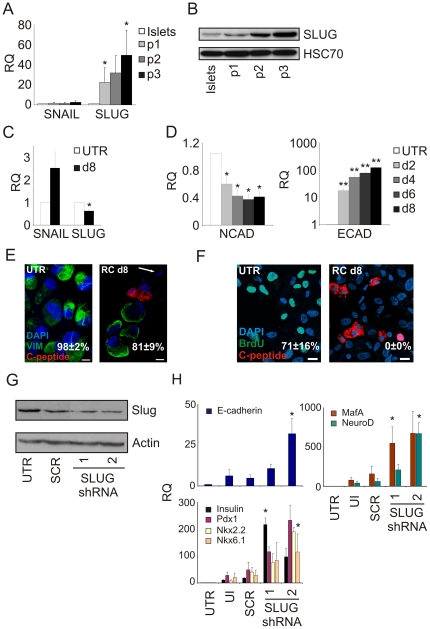
BCD cell redifferentiation involves MET. **A,B:** Induction of SLUG expression during islet cell dedifferentiation. **A:** qPCR analysis of expanded islet cells at the indicated passages. Values are mean±SE relative to uncultured islets (n = 4 donors) and normalized to human RPLPO. *p<0.05. **B:** Immunoblotting analysis of protein extracted from uncultured islets and expanded islet cells at the indicated passage number. HSC70, heat-shock cognate 70. **C:** Downregulation of SLUG expression during islet cell redifferentiation. qPCR analysis of expanded islet cells at p6–7 following 8d treatment with RC. Values are mean±SE relative to untreated cells (n = 4 donors) and normalized to human RPLPO. *p<0.05. **D:** qPCR analysis of kinetics of changes in NCAD and ECAD transcripts in expanded islet cells at p5 during 8d treatment with RC. Values are mean±SE (logarithmic scale for ECAD) relative to untreated cells (n = 4 donors) and normalized to human RPLPO or GAPDH. *p<0.05; **p<0.01. **E:** Immunostaining analysis of expanded islet cells at p6 following 8d treatment with RC shows a decrease in the number of vimentin-positive cells (mean±SD, based on counting >400 cells from each of 3 donors), and mutually-exclusive expression of vimentin and C-peptide. Arrow points to a cell that may have already lost vimentin expression but has not activated yet insulin expression. Bar = 10 µm. **F:** Co-staining for C-peptide and BrdU in expanded islet cells at p5 following 8d treatment with RC. Percentages indicate the fraction of cells positive for BrdU among total cells, following BrdU incorporation in days 2–8 of the redifferentiation treatment. Data are mean±SD, based on counting 500 cells from each of 3 donors. Bar = 20 µm. **G:** Immunoblotting analysis of expanded islet cells at p5 transduced with lentiviruses expressing one of two SLUG shRNAs or a scrambled (SCR) shRNA. UTR, untreated cells. **H:** qPCR analysis of beta-cell transcripts in expanded islet cells at p5 transduced with lentiviruses expressing one of two SLUG shRNAs or a scrambled shRNA and treated with RC for 4 days. Values are mean±SE relative to untreated cells (n = 3–6 donors) and normalized to human RPLPO or GAPDH. *p<0.05, relative to SCR. UI, uninfected cells treated with RC.

qPCR analysis revealed a significant upregulation of both transcript and protein levels of the EMT effector SLUG (SNAI2) during dedifferentiation, and a small but significant downregulation during RC-induced redifferentiation (Figure 4A–C). In contrast, no significant changes were noted in expression of SNAIL (SNAI1) (Figure 4A,C) and TWIST (data not shown), two other transcription factors involved in EMT. Transcripts for the epithelial marker E-cadherin were significantly upregulated by RC, while those encoding the mesenchymal marker N-cadherin were significantly reduced (Figure 4D). In addition, while almost all expanded islet cells stained positive for the mesenchymal marker vimentin, upon redifferentiation C-pep^+^ cells were vimentin-negative, and the incidence of vimentin^+^ cells in the cell population decreased from 98±2% to 81±9% (Figure 4E). We also found cells negative for both markers (Figure 4E, arrow), which may represent cells that turned off vimentin expression but have not yet activated insulin expression. These data indicate that redifferentiated BCD cells transition from a mesenchymal to an epithelial phenotype.

Staining of the redifferentiated cells for Ki67 did not detect positive cells, suggesting that cell differentiation was accompanied with growth arrest (data not shown). To further validate this possibility, expanded islet cells were treated with RC in the presence of BrdU. While control cells grown in expansion medium readily incorporated BrdU, BrdU^+^ cells were not detected in cultures from 3 independent donors following RC treatment (Figure 4F). In addition, only rare cells (0.9±0.6% based on counting >500 cells in each of 3 donors) were apoptotic following the full course of RC treatment, as determined by TUNEL assay (data not shown).

To further explore the role of SLUG downregulation in BCD cell redifferentiation, we employed two SLUG shRNAs to reduce SLUG expression beyond the small reduction (38±6%) induced by RC. SLUG shRNA reduced SLUG protein levels by ∼70% (Figure 4G). When combined with RC, two different SLUG shRNAs stimulated beta-cell transcript levels several fold, compared with scrambled shRNA (Figure 4H), confirming the importance of SLUG downregulation for BCD cell redifferentiation.

### Lineage-traced cells give rise to cells expressing other islet hormones

**Figure 5:**
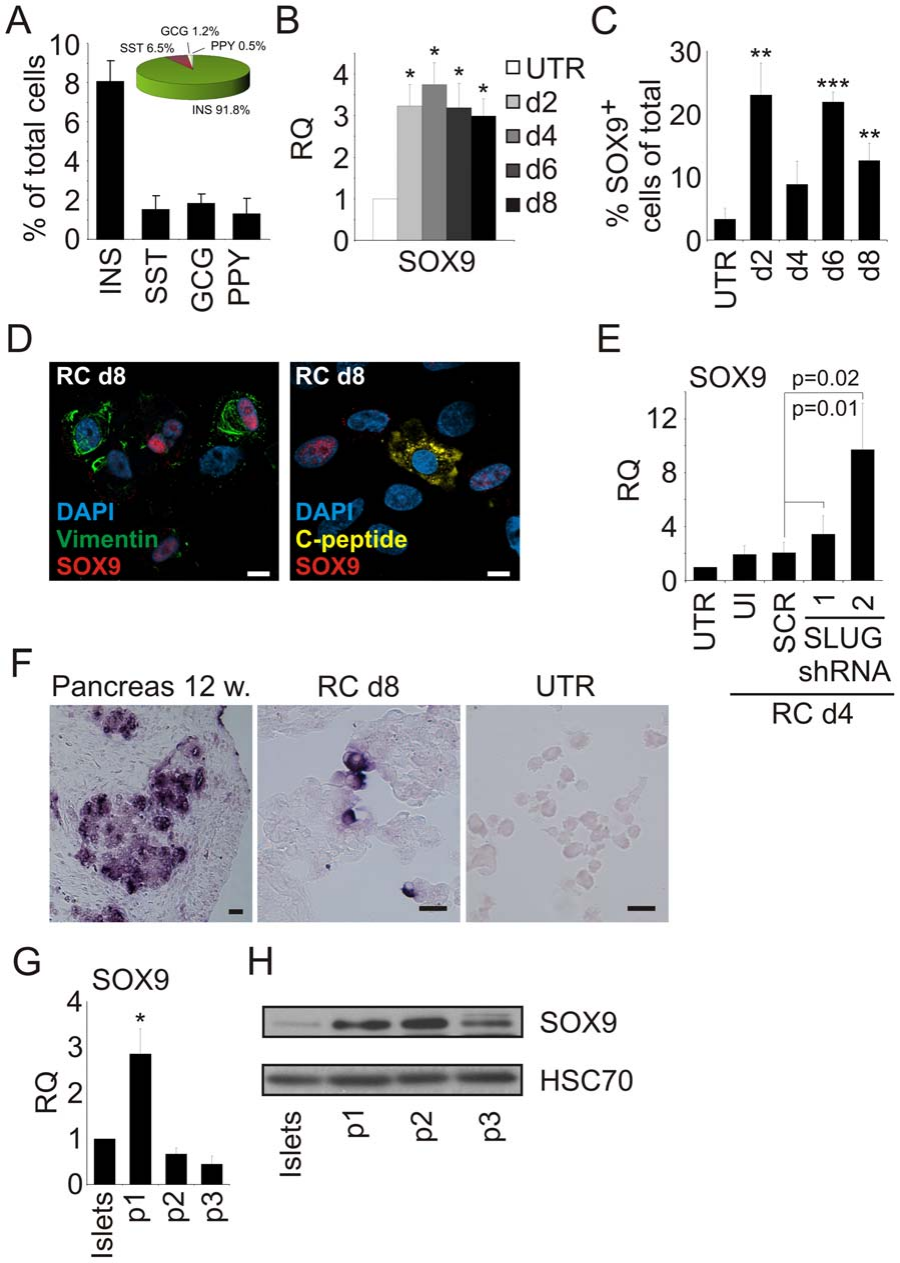
BCD cell redifferentiation involves expression of pancreas- and islet-progenitor transcription factors SOX9 and NGN3, respectively. **A:** RC treatment of expanded islet cells (p6) results in generation of hormone-expressing cells other than insulin. Cells were treated with RC for 8 days and stained with antibodies for the indicated hormones (insulin-producing cells were identified by staining for C-peptide). Values are mean±SE (n = 4 donors), based on counting >500 cells from each donor. The inset pie-chart depicts the percent of cells positive for each hormone among a total of 1736 hormone-positive eGFP^+^ cells scored in parallel experiments with lineage-traced cells from 6 donors. **B–D:** SOX9 activation during redifferentiation. Islet cells at p5 were treated with RC for 8 days. **B:** qPCR analysis of kinetics of upregulation of SOX9. Values are mean±SE, relative to untreated cells (d0) (n = 4 donors), and normalized to human RPLPO or GAPDH. *p<0.05. **C:** Quantitation of SOX9^+^ cells by immunofluorescence analysis. Values are mean±SD, based on counting >500 cells from each of 3 donors at each time point. **p≤0.01, ***p≤0.001, relative to untreated cells (d0). **D:** Left, Immunofluorescence analysis detects cells stained for vimentin or SOX9 alone, as well as double-positive cells. Right, C-peptide and SOX9 staining is mutually exclusive. Bar = 10 µm. **E:** qPCR analysis of SOX9 transcripts in expanded islet cells at p5 transduced with lentiviruses expressing one of two SLUG shRNAs or a scrambled shRNA and treated with RC for 4 days. Values are mean±SE relative to untreated cells (n = 4–6 donors) and normalized to human RPLPO or GAPDH. UI, uninfected cells treated with RC. **F:** NGN3 is activated during redifferentiation. In-situ hybridization analysis of NGN3 mRNA expression in human fetal pancreas cells (left panel) and expanded islet cells at p5 following 8d treatment with RC treated islet cells (right panel). Bar = 25 µm. **G–H:** Transient activation of SOX9 during islet cell dedifferentiation. **G:** qPCR analysis of expanded islet cells at the indicated passages. Values are mean±SE relative to uncultured islets (n = 3 donors) and normalized to human RPLPO. *p<0.05. **H:** Immunoblotting analysis of protein extracted from uncultured islets and expanded islet cells at the indicated passage number.

In addition to losing insulin expression, expanded islet cells are devoid of cells expressing glucagon (GCG), somatostatin (SST), and pancreatic polypeptide (PPY) (data not shown). RC treatment resulted in appearance of immunostaining for each of these hormones in ∼2% of the treated cells (Figure 5A). Importantly, no hormone co-expression was detected. To determine the origin of these cells, eGFP-labeled expanded cells were treated with RC and co-stained for eGFP and the four islet hormones. As seen in Figure 5A, the majority (91.8%) of eGFP^+^ cells which activated islet hormone expression were C-pep^+^. However, a small percent expressed instead one of the other islet hormones, most notably SST (6.5%). These analyses suggested that at least part of the cells expressing islet hormones other than insulin were derived from BCD cells, raising the possibility that redifferentiating BCD cells transited through an islet progenitor-like stage. Analysis of expanded islet cells for transcripts of pancreas- and islet-progenitor cell transcription factors did not detect expression of PTF1A, TCF2, and PAX4 (Ct >40), and showed low but detectable levels of SOX9, FOXA2, and ARX transcripts (data not shown). Following RC-induced redifferentiation, expression of some of these factors was significantly induced. Thus, SOX9 transcripts were upregulated 4-fold (Figure 5B), and >20% of the cells became SOX9^+^ within 2 days of RC treatment (Figure 5C). In addition, FOXA2, PAX4, and ARX were also significantly upregulated during this treatment (Figure S3). Cells co-expressing SOX9 and vimentin could be detected, however SOX9 and C-peptide expression was mutually exclusive (Figure 5D), suggesting a transient activation of SOX9 during redifferentiation and MET. SOX9 transcript levels were stimulated 5-fold by SLUG shRNA (Figure 5E), suggesting that SOX9 expression is downstream of MET. CK19- or amylase-positive cells could not be detected following RC treatment (data not shown), suggesting that the expanded islet cells did not give rise to duct- or acinar-like cells.

Expression of *NGN3*, a marker of islet progenitor cells, could not be reproducibly detected by qPCR during redifferentiation. However, we could detect a significant increase in transcripts of *INSM1* (Figure S4), a direct target of NGN3 [24]. RNA in-situ hybridization revealed rare *NGN3* ^+^ cells on day 2 of the RC treatment, and increasing numbers on days 4, 6, and 8 (Figure 5F), suggesting a transition through a NGN3^+^ stage during BCD cell redifferentiation. No *NGN3* ^+^ cells were detected in expanded islet cells untreated with RC (Figure 5F). Taken together, these findings suggest that BCD cell redifferentiation proceeds through an islet-progenitor-like stage, which may allow a low rate of differentiation into other islet cell types, in addition to insulin-producing cells, in particular the developmentally-related SST^+^ cells. We also detected transient SOX9 activation during the adaptation of primary islet cells to proliferation in culture (Figure 5G,H), suggesting that cell dedifferentiation also transits through a progenitor-like stage.

## Discussion

**Figure 6:**
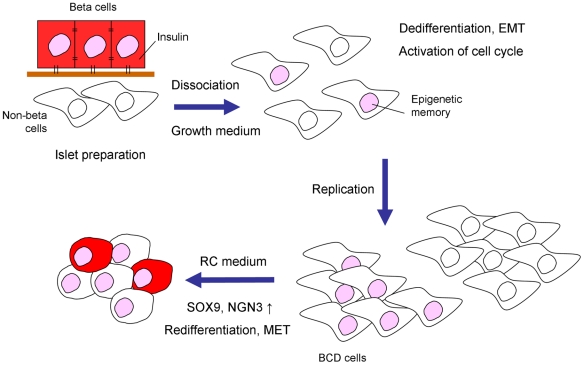
Scheme of beta-cell expansion and redifferentiation. Human islet preparations are dissociated into single cells and cultured in growth medium. Beta-cell dissociation from epithelial structure, and exposure to growth factors, induce EMT and dedifferentiation, but BCD cells maintain epigenetic memory. All cell types in the mixed islet cell culture, of which ∼45% are BCD cells, are induced to replicate. Following exposure to RC treatment, ∼25% of BCD cells undergo redifferentiation, which is associated with MET and activation of SOX9 and NGN3 expression. De-novo differentiation of non-BCD cells does not contribute significantly to generation of insulin-producing cells.

Our results present an approach for expansion of insulin-producing cells from adult human islets in two stages, the first involving expansion of the mixed islet cell population, including ∼45% BCD cells, for up to 16 population doublings, followed by a second stage of specific redifferentiation of BCD cells within the expanded islet cell population (Figure 6). RC treatment achieved a remarkably reproducible differentiation in cells from all human donors tested. These conditions induced a profound phenotypic change in the expanded cells, involving activation and shut-off of multiple genes. Lineage tracing suggests that the predominant source of newly-generated insulin-producing cells in these cultures is redifferentiation of BCD cells. These findings demonstrate for the first time that expanded dedifferentiated beta cells can be induced to redifferentiate in culture. Although we can not exclude a small contribution of other cell types to the C-pep^+^ cells generated by RC treatment, the percent of label-positive cells among C-pep^+^ cells is within the range of labelling efficiency, supporting the likelihood that the bulk of differentiation represents BCD cell redifferentiation. The mixed islet cell cultures may contain cells expanded from MSCs originally present in the islet preparation [25]; however we could not induce insulin expression with RC in non-BCD mesenchymal cell types, such as fibroblasts or BM-MSCs. The specific redifferentiation capacity of BCD cells may be explained by a permissive epigenetic state (“epigenetic memory”). Thus, beta-cell genes may be poised for expression, however their lack of transcription may reflect an abnormal balance between transcription activators and repressors, and/or other chromatin modifications. Culture conditions in the redifferentiation medium may restore the proper balance without requirement for extensive epigenetic modifications.

Upon transfer to RC, BCD cells are depleted of growth factors present in the serum of the expansion medium, resulting in growth arrest. However, growth arrest by itself is not sufficient for induction of redifferentiation in expanded islet cells, as suggested by overexpression of the cell cycle inhibitor p57, which induced growth arrest without differentiation (Bar et al., unpublished results). The absence of serum also stimulates cell aggregation into islet-like clusters, which likely contributes to formation of cell conformation and cell-to-cell contacts required for BCD cell redifferentiation. We previously observed that proliferation of sorted BCD cells depended on factors secreted by non-BCD cells in the islet cell culture [11]. It is possible that other cell types present in the islet cell culture also provide components (e.g ECM, secreted factors) needed for redifferentiation of BCD cells. Redifferentiation of purified BCD cells, in the absence of non-BCD cells, will help address this possibility.

BCD cell redifferentiation involves MET, as judged by changes in gene expression, and by stimulation of redifferentiation upon inhibition of the EMT effector SLUG. The mechanistic correlation between MET and redifferentiation remains to be elucidated. It is possible that restoration of ECAD expression reinstates the normal cell-to-cell contacts needed for activation of beta-cell genes. Alternatively, transcription factors involved in EMT may act as repressors of beta-cell gene expression.

Treatment with RC induced redifferentiation of up to 25% of all BCD cells at p5–7. This represents a net 8–32 fold increase in the number of insulin-producing cells, compared to uncultured islets. Improved redifferentiation conditions may extend the redifferentiation capacity to later passages, and increase the fraction of BCD cells undergoing redifferentiation, thus allowing generation of a larger mass of insulin-producing cells. High-throughput screening of compound libraries may identify agents which can further stimulate redifferentiation of the expanded BCD cells. Improved redifferentiation protocols, followed by sorting of the insulin-producing cells generated, will allow functional analyses in vivo, in immunodeficient mice. In addition to improving the redifferentiation protocol, extension of the expansion capacity beyond the ∼16 population doublings obtained in our current expansion conditions may be of potential value for generation of higher numbers of insulin-producing cells.

Generation of SST^+^ cells from lineage-traced cells suggests that redifferentiation involves BCD cell transition through an islet progenitor-like stage. Activation of expression of transcription factors characteristic of islet progenitor cells during redifferentiation, including SOX9, FOXA2, PDX1, NGN3, PAX4, and ARX, supports this possibility. Nevertheless, the predominant hormone-producing cell type generated from BCD cells by RC treatment is insulin-positive cells (91.8%), followed by SST^+^ cells (6.5%), and a small fraction of GCG^+^ and PPY^+^ cells (1.2% and 0.5%, respectively). It is intriguing to speculate that the epigenetic memory directs these transitory islet progenitor-like cells back into their cell type of origin, with some “leakage” into the developmentally-related SST^+^ cells. This culture system may provide an attractive model of human islet progenitor cell development, in which the cascade of transcription factor activation elucidated from mouse gene knockout models can be verified. Lineage tracing using progenitor cell gene promoters to drive Cre expression will be required to prove the transition through a progenitor-like stage during redifferentiation.

A recent paper presented evidence for expression of insulin in rare human pancreas multipotent stem cells [26]. These findings raise the possibility that our lineage tracing system labels these cells, in addition to bona-fide beta cells, and therefore the generation of insulin-positive cells observed in this study could be attributed to de-novo differentiation of such stem cells, rather than to redifferentiation. While we can not exclude contribution of these cells, they are unlikely to be the major source of the hormone-expressing cells generated in our differentiation culture conditions. Our previous work documented a widespread proliferation of eGFP^+^ cells starting during the early days of islet cell culture [11], which is inconsistent with expansion of a rare cell type. In addition, a multipotent pancreatic stem cell would be expected to give rise to all pancreatic cell types, including duct and acinar cells, which could not be detected following RC treatment. Thus, rather than reflecting expansion of rare pancreas multipotent stem cells, our data is consistent with transition of redifferentiation through an islet progenitor-like stage, suggesting that redifferentiation may recapitulate some aspects of islet development.

Our findings suggest that ex-vivo expansion of adult human islet cells is a promising approach for generation of insulin-producing cells for transplantation, as well as research, toxicology studies, and drug screening.

## Supporting Information

Figure S1 **Specificity of RIP-CreER transgene expression in beta cells.** Human islet cells were transduced with the RIP-CreER lentivirus vector and co-stained for Cre protein and the 4 pancreatic islet hormones 2–3 d post infection (insulin^+^ cells were identified by staining for C-peptide). Values are mean±SD (n = 3 donors; based on counting 400 cells from each donor).
(TIF)

Figure S2 **Islet cell differentiation decreases with cell passaging.** Insulin transcript levels in isolated islets, expanded islet cells, and redifferentiated cells at the indicated passages, were quantified by qPCR analysis. Results are mean±SE relative to uncultured islets and normalized to human RPLPO (n = 3–6 donors).
(TIF)

Figure S3 **Kinetics of upregulation of islet progenitor cell genes during RC treatment.** qPCR analysis of expanded islet cells at p5 treated with RC for the indicated number of days. Values are mean±SE, relative to untreated cells (d0) (n = 4 donors), and normalized to human GAPDH, *p<0.05, **p≤0.01, ***p≤0.001.
(TIF)

Figure S4 **Upregulation of INSM1 following RC treatment.** qPCR analysis of INSM1 transcripts in expanded islet cells at p5 following 8d treatment with RC. Values are mean±SE relative to untreated cells (n = 4 donors) and normalized to human RPLPO.
(TIF)
