# Supplementary figures and images for: Insulin-Producing Cells Generated from Dedifferentiated Human Pancreatic Beta Cells Expanded In Vitro
Source: PLoS One. 2011 Sep 30;6(9):e25566. doi: 10.1371/journal.pone.0025566 (PMC3184150; doi:10.1371/journal.pone.0025566)

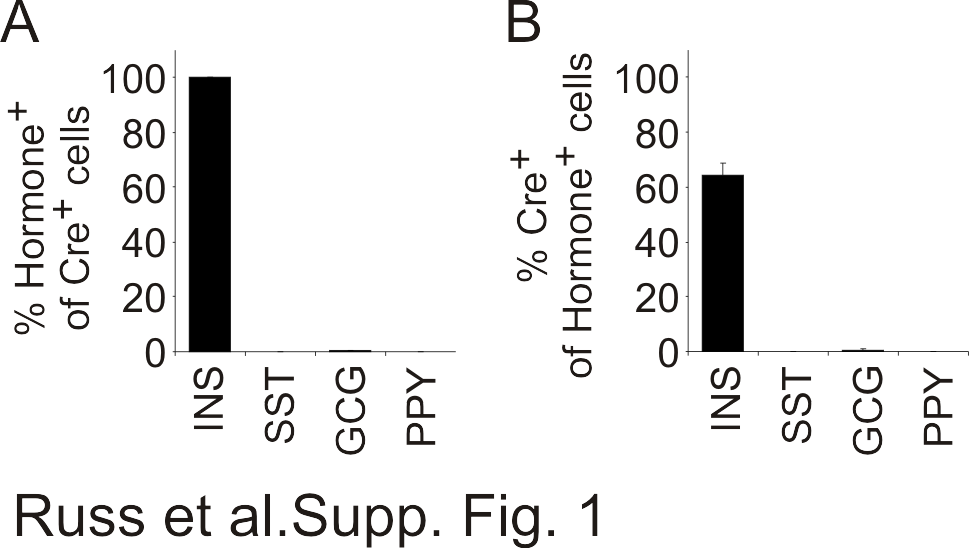

Supplement: Figure S1 — Specificity of RIP-CreER transgene expression in beta cells. Human islet cells were transduced with the RIP-CreER lentivirus vector and co-stained for Cre protein and the 4 pancreatic islet hormones 2–3 d post infection (insulin+ cells were identified by staining for C-peptide). Values are mean±SD (n = 3 donors; based on counting 400 cells from each donor). (TIF) [file pone.0025566.s001.tif]

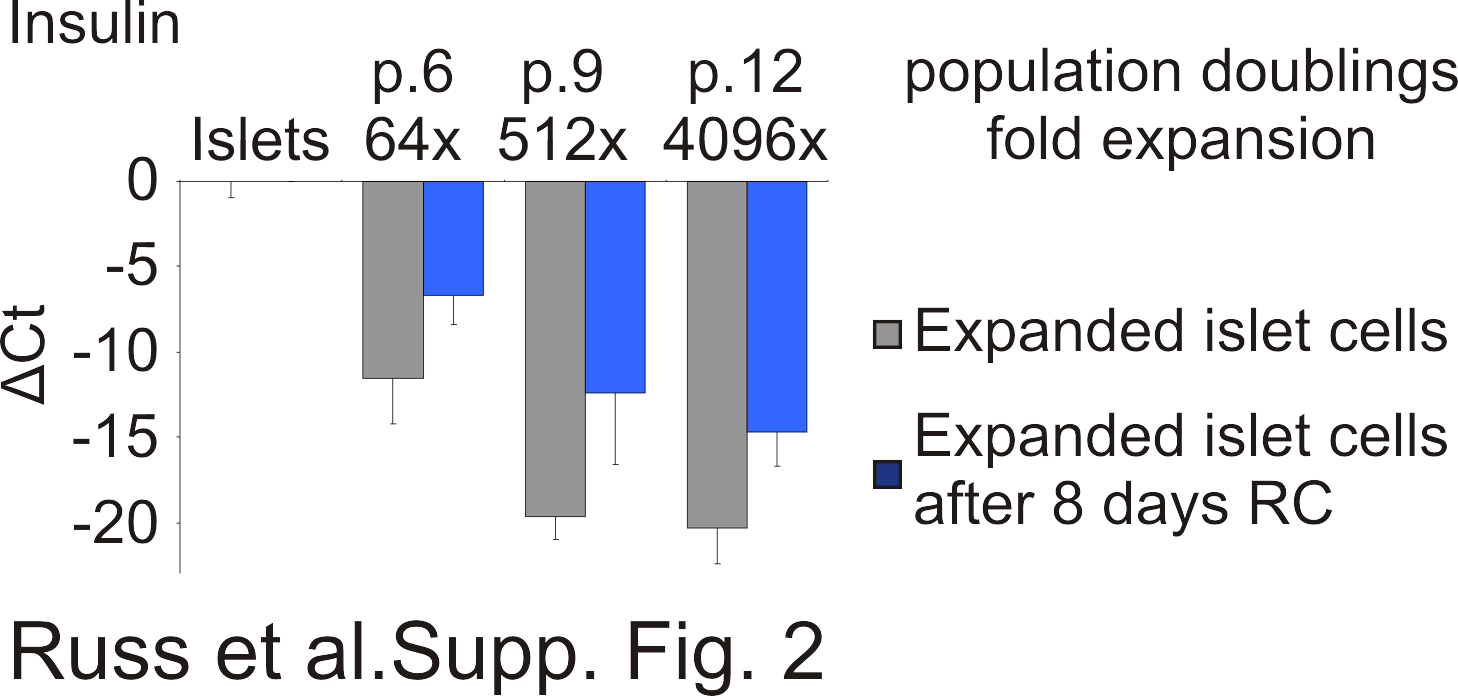

Supplement: Figure S2 — Islet cell differentiation decreases with cell passaging. Insulin transcript levels in isolated islets, expanded islet cells, and redifferentiated cells at the indicated passages, were quantified by qPCR analysis. Results are mean±SE relative to uncultured islets and normalized to human RPLPO (n = 3–6 donors). (TIF) [file pone.0025566.s002.tif]

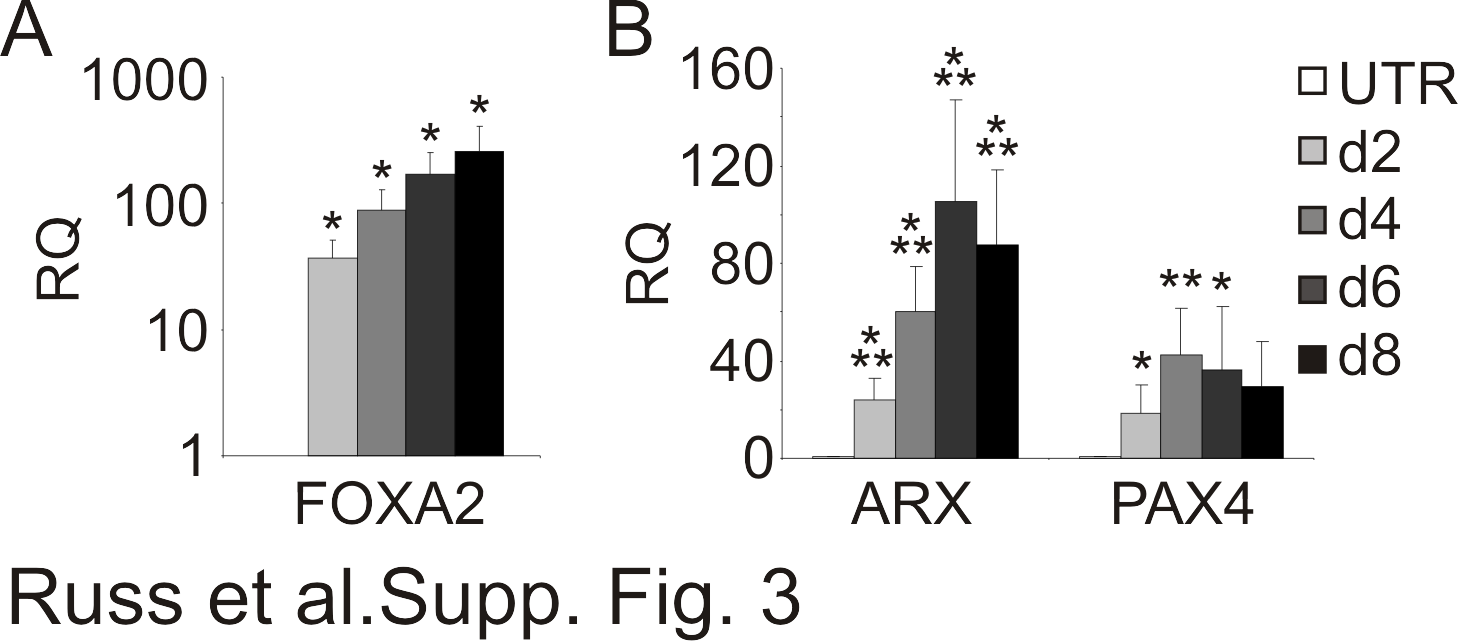

Supplement: Figure S3 — Kinetics of upregulation of islet progenitor cell genes during RC treatment. qPCR analysis of expanded islet cells at p5 treated with RC for the indicated number of days. Values are mean±SE, relative to untreated cells (d0) (n = 4 donors), and normalized to human GAPDH, *p<0.05, **p≤0.01, ***p≤0.001. (TIF) [file pone.0025566.s003.tif]

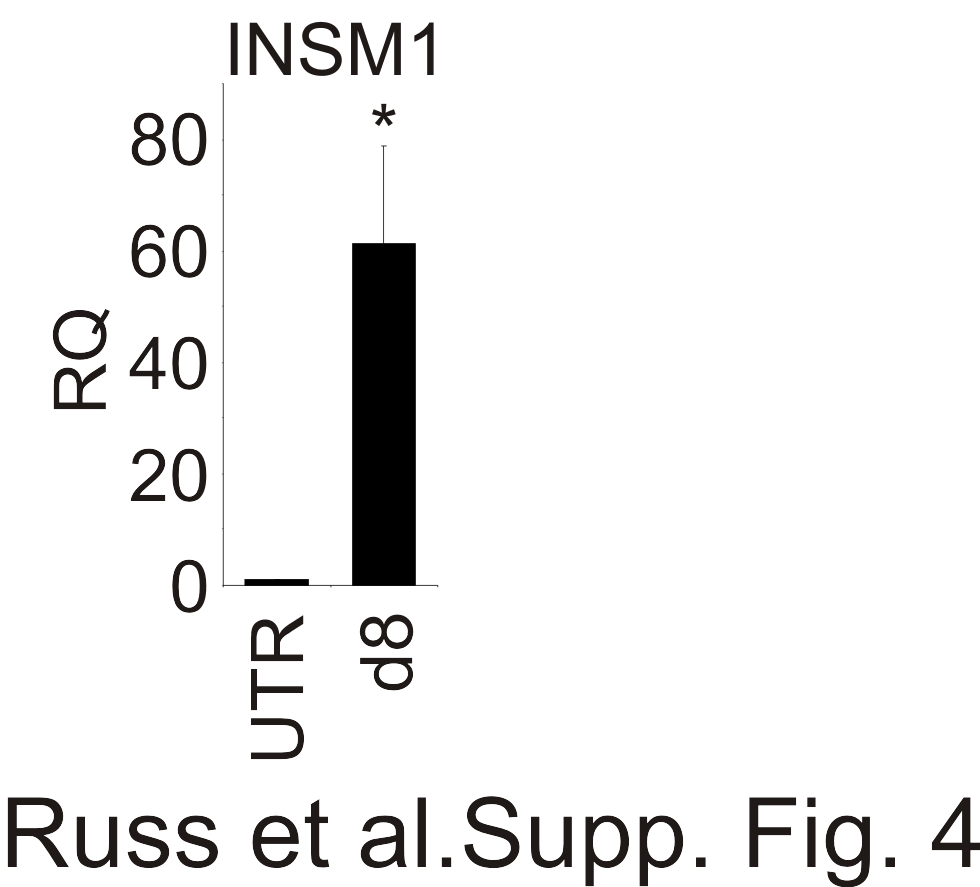

Supplement: Figure S4 — Upregulation of INSM1 following RC treatment. qPCR analysis of INSM1 transcripts in expanded islet cells at p5 following 8d treatment with RC. Values are mean±SE relative to untreated cells (n = 4 donors) and normalized to human RPLPO. (TIF) [file pone.0025566.s004.tif]
